# Efficacy and safety of *Qing-Feng-Gan-Ke Granules* in patients with postinfectious cough: study protocol of a novel-design phase III placebo-controlled, double-blind randomized trial

**DOI:** 10.1186/s12906-015-0812-3

**Published:** 2015-08-19

**Authors:** Wei Liu, Hongli Jiang, Ruiming Zhang, Faguang Jin, Liangji Liu, Youyu Long, Liying Cui, Suyun Li, Yunqing Zhong, Bing Mao

**Affiliations:** Department of Integrated Traditional and Western Medicine, West China Hospital of Sichuan University, 37 Guoxue Lane, Chengdu, Sichuan Province 610041 China; Department of Respiratory and Critical Care Medicine, Tangdu Hospital of The Fourth Military Medical University, 1 Xinsi Road, Xi’an, Shaanxi Province 710038 China; Department of Respiratory Medicine, Affiliated Hospital of Jiangxi University of Traditional Chinese Medicine, 445 Bayi Avenue, Nanchang, Jiangxi Province 330006 China; Department of Respiratory Medicine, The Second Affiliated Hospital of Tianjin University of Traditional Chinese Medicine, 816 Zhenli Street, Tianjin, 300150 China; Department of Respiratory Medicine, Affiliated Hospital of Inner Mongolia Medical University, 1 North Tongdao Street, Hohhot, Inner Mongolia 010000 China; Department of Respiratory Diseases, The First Affiliated Hospital of Henan University of Traditional Chinese Medicine, 19 Renmin Road, Zhengzhou, Henan Province 450000 China; Department of Respiratory Medicine, The First Affiliated Hospital of Guangxi University of Traditional Chinese Medicine, 89-9 Dongge Road, Nanning, Guangxi Province 530023 China

## Abstract

**Background:**

Postinfectious cough (PIC) is a common condition that affects millions of people worldwide every year. There is Western medicine for this condition but the treatment effect is often incomplete. Traditional Chinese medicine (TCM) has been increasingly prescribed for patients with PIC. Preliminary trials on *Qing-Feng-Gan-Ke-Granules* (QFGKG) conveyed promising results in treating PIC. This protocol describes an ongoing phase III randomized controlled clinical trial, designed according to a novel methodology of “one study, one primary outcome”, with the objective of evaluating the efficacy and safety of QFGKG in patients suffering from PIC.

**Methods/Design:**

This is a multicenter, phase III, randomized, double-blind, parallel-group, placebo-controlled clinical trial, comprising two simultaneously conducted study parts, part A and part B, intending to investigate two primary outcomes, *i.e.* time to cough resolution and cough symptom score, respectively. A total of 480 patients, aged 18 to 65 years, who complain of an ongoing persistent cough that has been lasting ≥3 weeks, will be recruited from six participating sites and then randomized to receive QFGKG 12.0 g twice daily or placebo 12.0 g twice daily. Each part will enroll 240 patients, with 180 patients being allocated to the QFGKG group and 60 to the placebo group.

**Discussion:**

Although traditional Chinese medicine is a structured intervention that has shown some promise in treating persistent cough, existing unconvincing evidence has noted limitations. This is a rare well-designed and rigorously-controlled, randomized, double-blind trial to evaluate the effects and safety of a Chinese herbal medicine in patients with postinfectious cough, providing tangible benefits for clinical research. Results of this trial are inclined to be conjectured as more truthful by implementing separate study parts that specifically estimate exclusive primary outcome. It will not only provide robust clinical evidence on the efficacy and safety of QFGKG for postinfectious cough, but will also provide a critical piece of information on the availability and superiority of a novel methodology for future clinical trials. The current trial is ongoing with recruitment of the predetermined number of patients being in progress.

**Trial registration:**

The two parts of this trial were separately registered with the Chinese Clinical Trial Registry: ChiCTR-TRC-13003278 (part A); and ChiCTR-TRC-13003337 (part B).

## Background

Cough is the most common symptom presenting to primary healthcare services. Postinfectious cough (PIC) is considered as one of the top causes of subacute cough, which occurs in 11 to 25 % of patients with upper respiratory tract infection (URTI) [1, 2]. It involves a persistent cough lasting ≥3 weeks or even months [3] (but normally < 8 weeks) secondary to the acute symptoms of a common cold or upper respiratory tract infection (URTI) [4, 5]. Although PIC is always self-limited and is not associated with severe debilitation or mortality, persistent cough may impair the quality of daily life [4]. Therefore, many patients with PIC are frequently impelled to seek medical help [6].

Aside from pertussis, however, despite the fact that many pathogenic agents including respiratory viruses (particularly respiratory syncytial virus, adenoviruses, parainfluenza, and influenza), M pneumoniae and Chlamydophila pneumoniae have been implicated in children [4, 5, 7], the role of infectious aetiology in PIC in adults is rarely confirmed till now [8]. Poor knowledge on the pathogenesis of PIC, which is supposed to be frequently multifactorial [2, 8–10], results in a diagnostic challenge for physicians. Patients are constantly investigated for other causes of persistent cough [6]. Therapy with antibiotics has no role, as there is no evidence that bacterial infection plays a role in adult patients [4, 5]. Cysteinyl leukotrienes were considered to be involved in the pathogenesis of PIC in recent studies, however, montelukast failed to effectively treat PIC in a latest randomized trial [11], leading to a controversy on its role in PIC. Therefore, PIC can be a troublesome problem for physicians and patients alike [6]. Currently available therapies, including inhaled ipratropium, inhaled or oral corticosteroids, central acting antitussive agents such as codeine and dextromethorphan, can only be used for symptom relief [4–7, 12, 13]. They have not been proved to be effective in reducing the cough duration or improving the quality of life [4]. And above all, a range of adverse outcomes including blurred vision, confusion, difficulty in urination, drowsiness or dizziness, body rash/itching, nausea or vomiting, constipation, diarrhea, sedation, nervousness are elicited occasionally [14–16]. For these reasons, complementary and alternative medicine is becoming more and more popular in patients with PIC, such as a combination of honey and coffee [6] as well as traditional herbal medicines.

The use of traditional Chinese medicine (TCM) as a complementary and alternative therapy for cough can be dated back to thousands of years ago. People in China who suffer from a persistent cough always give first priority to TCM. With an increasing number of TCM formulas developed for clinical use, an overview to facilitate the discussion of its pros and cons is warranted. A recent comprehensive systematic review of randomized controlled trials demonstrated potential curative effects and reassured the safety of TCM in the management of PIC [17]. Unfortunately, due to the undesirable quality of included studies, the definite effect of TCM turned to be inconclusive in this review. Therefore, a rigorously designed randomized trial becomes imperative.

TCM offers a holistic vision on disease, and syndrome differentiation is the cornerstone of TCM prescription. Differentiation means comprehensive analysis, and syndrome refers to symptoms and signs. Differentiation of syndromes implies that the patient’s symptoms and signs collected by the four diagnostic methods (inspection, auscultation ant olfaction, inquiry, pulse-taking and palpation) are analyzed and summarized to identify the etiology, nature and location of a disease, thereby determining what syndrome the disease belongs to. Based on both clinical experience and literature review, “pathogenic-wind invading lung syndrome” is considered as the most common TCM syndrome in patients with PIC [17]. TCM patent drug *Qing-Feng-Gan-Ke-Granules* (QFGKG), designed on the basis of a classic formula, comprises four widely used herbal plants (*Ephedra sinica Stapf*, *Sinomenium acutum*, *Stemona japonica (Blume) Miq.*, and *Aster tataricus L.f.*). It has been shown that QFGKG is very effective in dispelling wind and relieving cough and is specifically appropriate for treating “pathogenic-wind invading lung syndrome”. Unpublished preliminary phpartacodynamics studies in rats showed that QFGKG could significantly alleviate sulfur dioxide-induced cough and ammonium hydroxide-induced cough, increase the tracheal phenolsulfonphthalein excretion, improve the peritoneal capillary permeability reduced by acetic acid, relieve asthma caused by acetyl choline and histamine inhalation, inhibit carrageenin-induced toes swelling, suppress dinitrochlorobenzene-induced delayed type hypersensitivity, which suggests its antitussive, anti-inflammatory and immunosuppressive properties. Subsequent clinical studies investigated its efficacy and feasibility in individuals with PIC [18, 19]. In addition, a previous multi-center phase II trial [20] investigated the dose-effect relationship and dose-safety relationship of QFGKG, with primary outcomes of time to cough relief and time to cough resolution, as well as secondary outcomes of cough symptom score, cough recovery rate, TCM curative effect, and Chinese version of cough-specific quality of life questionnaire (CQLQ) score [21]. In this phase II trial, 180 patients were enrolled and randomly assigned to receive high dose of QFGKG (12.0 g, twice daily), or low dose of QFGKG (6.0 g, twice daily) plus dummy QFGKG (6.0 g, twice daily), or dummy QFGKG (12.0 g, twice daily) alone. Great improvements in all outcomes, especially the time to cough resolution and cough symptom score, were observed in the high dose group. To the best of our knowledge, the phase II study was the first well-designed placebo-controlled clinical trial that reported TCM as a remedy for PIC, and its resultful conclusions provide foundations for the current phase III trial.

“One study, one primary outcome”, a novel methodology for clinical trial design, was published by China Food and Drug Administration (CFDA). The core of this concept lies in the supposition that study results will be more credible if the whole study is single-mindedly and exclusively designed for the unique outcome of main interest. Moreover, the SPIRIT guidance suggests a minimum number of primary outcomes in clinical trials because multiple primary outcomes can introduce problems with multiplicity, selective reporting, and interpretation when there are inconsistent results across outcomes [22]. Thus, although this design has not been widely implemented in clinical trials, we prefer to conduct a tentative trial in an attempt to provide some practical information.

The objective of this trial is to demonstrate the superior efficacy and safe of QFGKG over placebo in adult individuals with PIC by addressing the following hypotheses: (1) That QFGKG will significantly shorten the cough duration in patients with PIC; (2) That QFGKG will significantly relieve the main clinical symptoms in patients with PIC; (3) That QFGKG will increase the quality of life in patients with PIC; (4) That QFGKG is safe and will not result in any serious side effects; (5) That the novel design methodology of “one study, one primary outcome” is feasible and desirable in clinical trials.

## Methods/Design

### Study designs

**Fig. 1:**
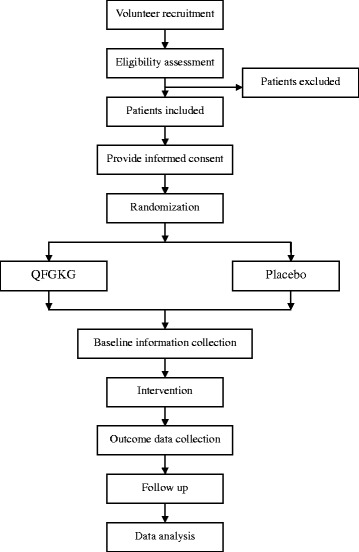
Flow chart of study process

This trial is designed as a randomized, double-blind, placebo-controlled, parallel, phase III, superiority trial, with allocations stratified by participating centers and randomization performed as block at a ratio of 3:1, adhering to the recommendation of “CONSORT statement” [23] and “SPIRIT statement” [24]. As to the allocation ratio, generally speaking, for a superiority trial using placebo as a control intervention, 3:1 is recommended by Provisions for Drug Registration (http://www.sda.gov.cn/ypzcgl/ml.htm), taking into full consideration of statistical significance, ethical requirement and risk-cost relationship. This trial consists of two simultaneously conducted study parts (part A and part B), currently involving six participating centers across China: West China Hospital of Sichuan University, Tangdu hospital of The Fourth Military Medical University, Affiliated Hospital of Jiangxi University of TCM, the Second Affiliated Hospital of Tianjin University of TCM, Affiliated Hospital of Inner Mongolia Medical University, and the First Affiliated Hospital of Henan University of TCM. These centers were defined primarily geographically. In each participating center, a chief investigator will be appointed to be responsible for the whole clinical process (subject recruitment, intervention, follow-up, and data collection), adhering to the study protocol. All personnel involved in this trial will be trained prior to trial initiation. Training program is about study protocol and relevant skills, especially the skill of TCM syndrome differentiation. Staff placement and individual responsibility will also be elaborated in the training session. Besides, investigator’s brochure developed by the sponsor to facilitate the smooth running of the trial will also be provided. This protocol is designed largely depending on the results of previous phase II trial. Both study parts have been registered with Chinese Clinical Trial Registry (http://www.chictr.org). This protocol has two amendments. The schematic diagram and flow chart of study process are shown in Tables 1, 2 and Fig. 1.

**Table 1:** Schematic diagram of part A

Phase	Supervision period			
Time-point	Baseline	Visit 1	Visit 2	Visit 3
Day	0	8 ± 1	15 ± 2	Follow-up
Basic history collection	×			
Informed consent	×			
Demographics	×			
Enrollment based on the inclusion/exclusion criteria	×			
Coexisting diseases or symptoms	×			
Concomitant treatments		×	×	
Efficacy evaluation				
Cough symptom score	×	×	×	×
TCM syndrome score	×	×	×	
Safety evaluation				
Vital signs	×	×	×	
Blood routine and urine routine	×		×	
Liver function (ALT, AST, STB, ALP, γ-GT )	×		×	
Renal function (Cr, BUN)	×		×	
ECG	×		×	
Adverse events		×	×	
Screening tests				
CR	×			
BPT	×			
UPT (women of childbearing age)	×			
Other steps				
Randomization allocation	×			
Drug distribution	×			
Remaining drug count			×	
Patient record card distribution/recycling	×		×

**Table 2:** Schematic diagram of part B

Phase	Supervision period			
Time-point	Baseline	Visit 1	Visit 2	Visit 3
Day	0	6 ± 1	11 ± 1	Follow-up
Basic history collection	×			
Informed consent	×			
Demographics	×			
Enrollment based on the inclusion/exclusion criteria	×			
Coexisting diseases or symptoms	×			
Concomitant treatments		×	×	
Efficacy evaluation				
Cough symptom score	×	×	×	×
Cough VAS score	×	×	×	
TCM syndrome score	×	×	×	
CQLQ	×		×	
Safety evaluation				
Vital signs	×	×	×	
Blood routine and urine routine	×		×	
Liver function (ALT, AST, STB, ALP, γ-GT )	×		×	
Renal function (Cr, BUN)	×		×	
ECG	×		×	
Adverse events		×	×	
Screening tests				
CR	×			
BPT	×			
UPT (women of childbearing age)	×			
Other steps				
Randomization allocation	×			
Drug distribution	×			
Remaining drug count			×	
Patient record card distribution/recycling	×		×

### Study participants

A total of 480 patients will be recruited and averagely assigned to each part. Each center will recruit an equal number of patients; 60 patients will be recruited at each individual center in part A and 80 patients will be enrolled at each individual center in part B. Patients, who are diagnosed as having PIC in one of the participating centers according to the guideline issued by the Chinese Medical Association [25], aged between 18 and 65 years, with a TCM syndrome being differentiated as “pathogenic-wind invading lung” based on the Guidelines for Clinical Research of New Chinese Medicine [26], are eligible for enrollment. Diagnosis criteria of PIC are presented in Table 3. Inclusion and exclusion criteria are listed in Table 4, and TCM syndrome diagnostic criteria, based on State Administration of Traditional Chinese Medicine guidelines [27], are elaborated in Table 5. Some discrepancies should be noticed in the inclusion criteria of two parts. Part A is designed to focus on the time when cough can be resolved completely, thus patients’ cough severity is relatively mild in order to yield more expected results. On the other hand, part B is designed to calculate the dynamic change in cough symptom score across four time-points (see Table 2), so the cough severity of patients recruited in this part is relatively severe for more obvious changes.

**Table 3:** Diagnosis criteria for postinfectious cough

● Cough that has been present following symptoms of an upper respiratory tract infection;
● Irritable dry cough or cough with small amounts of white mucus;
● Cough lasts for at least 3 weeks, but normally no longer than 8 weeks;
● Negative findings of chest radiograph;
● Negative results of bronchial provocation test and dilation test;
● Exclude other causes which could result in cough, such as upper airway cough syndrome(UACS), cough variant asthma(CVA), eosinophilic bronchitis(EB) and gastroesohageal reflux disease(GERC);
● Exclude severe pulmonary diseases including chronic obstructive pulmonary disease(COPD),lung cancer, pulmonary tuberculosis, et al.;
● Exclude hypertension that involves use of angiotensin converting enzyme inhibitor(ACEI) currently.

**Table 4:** Selection criteria for eligible patients

● Inclusion criteria for study part A	● Inclusion criteria for study part B
● Postinfectious cough	● Postinfectious cough
● Syndrome of “pathogenic-wind invading lung”	● Syndrome of “pathogenic-wind invading lung”
● Daytime cough symptom score ≥ 2 and/or nighttime cough symptom score ≥ 2	● Total cough symptom score ≥ 4
● Persistent cough ≥ 3 weeks and ≤ 6 weeks	● Persistent cough ≥ 3 weeks and ≤ 6 weeks
● Age 18-65 years old	● Age 18-65 years old
● Agreement to participate and provision of informed consent	● Agreement to participate and provision of informed consent
● Exclusion criteria	
● Body temperature > 37.3 °C	
● Use of any medication intended to treat PIC within one week before enrollment	
● ALT/AST > 1.5 times than the upper limit of normal reference values; Abnormal blood creatinine; Urine protein qualitative test > +; Blood leukocyte count < 3.0 × 10^9^/L or > 10.0 × 10^9^/L; and/or neutrophil percentage > 80%	
● History of any severe underlying diseases (*e.g.* cardiovascular, hematological, hepatic, renal); or other life-threatening disabling conditions and concomitant diseases (*e.g.* cancer, AIDS)	
● Pregnancy or potential pregnancy or lactation	
● Hypersensitivity or allergy to any component of the test drug	
● Current psychiatric disorders or legal disability	
● Current or previous admission to other investigational drug studies within 3 months before enrollment	
● Inability to complete the trial as decided by the investigators

**Table 5:** Diagnostic criteria for TCM differentiation of “pathogenic-wind invading lung syndrome”

Item	Symptoms and signs
*Primary symptom	persistent cough
Secondary symptoms	1) *throat itching, cough sensitivity to cold air, heat air or strange odors and/or;
	2) sticky and scanty sputum, dry throat and chest congestion
*Tongue picture	slight red tongue proper with/without red tip; thin white tongue coating
*Pulse condition	normal or floating pulse

* Items which must be satisfied for a diagnosis of TCM differentiation of “pathogenic-wind invading lung syndrome”

Patients must provide written informed consent before any study procedures occur. Participants would be excluded if they have any of the following conditions: 1) misdiagnosis; 2) poor drug compliance (taking < 80 % or > 120 % of the required dosage); 3) inability to complete the follow-up; 4) aggravations (such as dyspnea) that happen within 24 h after taking the test drug (on this occasion, patients will be treated with conventional therapies and considered as an invalid case, whose last data would be collected in Full Analysis Set (FAS)); 5) hypersensitivity or other severe adverse reactions; and 6) voluntary withdrawal. The trial will be terminated prior to its planned end date in the context of the following accidents: 1) protocol deviations; 2) inadvisable or flawed protocol; 3) compelling suspension forced by authoritative agencies; and 4) discontinuation decided by the trial sponsor (managerial or financial difficulties).

### Randomization and blinding

Eligible patients will be randomized in a ratio of 3:1 to QFGKG group or placebo group, with stratification by participating center. A centralized randomization will be employed. The random coding of each center as well as the allocation sequence are generated electronically using SAS 6.12 software. Assignments are concealed in sequentially prepared coded, sealed, opaque envelopes, in which the corresponding intervention group of the randomization list will be found. A professional statistician who is independent of intervention and data collection is appointed to choose appropriate block length to ensure concealment. A group of research assistants who are not involved in recruitment, intervention or assessment of outcomes will prepare the envelopes. Staff responsible for recruitment is not allowed to take part in the group allocation. Only after the former participant is enrolled and his/her baseline testing is completed, the next envelope could be opened by researchers responsible for allocation. Participants will be randomized to the intervention group or the control group in line with numbered envelope under instructions from drug managers at each center. Blind codes along with random seeds and block length will be recorded in duplicate and delivered to Good Clinical Practice (GCP) Centre of West China Hospital and the sponsor for safekeeping. Patients and all engaged personnel including the sponsor remain blind to allocation and intervention throughout the whole process. Placebo will be packaged with imitative similarity in appearance, smell and taste with QFGKG. However, personnel who are designated to prepare drug packaging and labeling will not be blinded. The prepared emergency code-break envelope that contains allocation information, and emergency measures as well as contact details of the responsible department will be distributed to study centers along with drugs. Patients reported side effects will be recorded in the case report forms (CRFs) detailedly by physicians at visit 1 and visit 2. If an unpredictable severe adverse event happens, the chief investigator is qualified to open the emergency envelope and guide physicians to treat the patient according to emergency measures. The patient must be withdrawn and be followed up until he/she recuperates. In this case, the chief investigator should report the case to hospital ethical committee and drug administration agency. The broken code and reasons for unblinding should be recorded on CRF. Meanwhile, severe adverse event report form (SAERF) will also be completed. During the revealing, allocation information must not be disclosed to the patient and any third party.

### Sample size

The latest edition of Provisions for Drug Registration issued by CFDA recommends a minimum number of 300 for subjects enrolled in the trial group in a phase III clinical trial. Sample size calculation of this trial results in 24 patients in total for part A (18 in the QFGKG group and 6 in the placebo group) and 56 patients in total for part B (42 in the QFGKG group and 14 in the placebo group), respectively. Obviously, the calculated patient number in the QFGKG group is much smaller than the recommended one. Given the above, we set the sample size according to the Provisions for Drug Registration. 480 patients in total would be included, with 360 being assigned to the QFGKG group and 120 being assigned to the placebo group, considering a maximal dropout rate of 20 %. Calculation assumed an 80 % power for a two-sided log-rank test at a 5 % significance level.

### Interventions

After enrollment, eligible patients will be randomly assigned to receive twice daily QFGKG 12.0 g or twice daily dummy QFGKG (placebo) 12.0 g. The use of placebo as a comparator in this trial largely depends on empirical evidence [17, 28, 29] and Guidelines for Clinical Research of New Chinese Medicine [26]. Besides, the absence of generally recognized therapy and self-healing tendency of PIC also support the use of placebo. QFGKG and placebo in this trial are manufactured by Baotou TCM Co., Ltd. The Latin names, English names and Chinese Pinyin of the four herbs used in QFGKG are listed in Table 6. Both within-group changes and between-group changes are assessed across three time-points during treatment. As to the treatment duration, a longer (14 days) treatment in part A is conducted for the sake of more cured cases, while a shorter treatment (10 days) in part B is enough for monitoring noticeable changes in cough symptom score. A two-day follow-up will be conducted for all patients. At the initial drug dispensing stage, patients are advised to strictly follow protocol instructions, which will be reiterated at each study visit. Participants are requested to genuinely report their daily dosage of drugs that have been actually taken and to return unused ones to physicians at each study visit. Drug using and returning will be recorded on CRF by physicians. Unused drugs will be finally collected and counted by drug managers and then be destroyed by the sponsor at the end of the trial. All previous and concomitant treatments, including drug and non-drug therapies, taken one week before and during the intervention will be recorded on CRF. Concomitant treatment for comorbidities such as hypertension, diabetes and other chronic conditions are permitted during the intervention. Whereas, the use of antibiotics, Western or other antitussives, and non-drug cough suppressing therapies such as acupuncture and cupping are strongly discouraged to prevent potential systemic effects. Participating centers are required to record dropout cases and reasons, and also side effects happen during the intervention.

**Table 6:** Latin name, English name and Chinese pinyin of the four herbs

Latin name	English name	Chinese pinyin
*Ephedra sinica Stapf*	Herba Ephedrae	Ma Huang
*Sinomenium acutum*	Caulis Sinomenii	Qing Feng Teng
*Stemona japonica (Blume) Miq.*	Radix Stemonae	Bai Bu
*Aster tataricus L.f.*	Aster tataricus	Zi Wan

### Outcome measures

#### Part A

##### Primary outcome

The primary outcome of part A is time to cough resolution, i.e. the time to the first day when cough completely disappears. Cough resolution is defined as both daytime cough symptom score nighttime cough symptom score graded as 0, for 48 h. If the total cough symptom score is classified as 0 in the two successive days, the previous day should be considered as the first day when cough completely disappears (Note: cough symptom score is used to assess cough symptoms within the last 24 h). For example, if the total cough symptom score of a patient is rated as 0 on the fifth day and the sixth day after intervention, then the time to cough resolution is regarded as five days.

##### Secondary outcomes

**Fig. 2:**
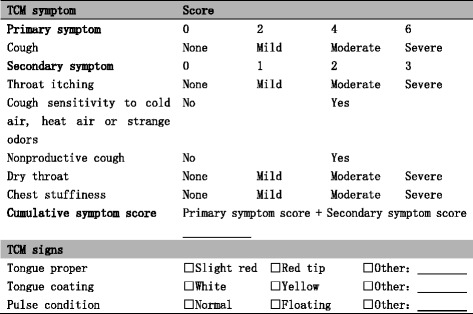
TCM symptom score and TCM signs

Secondary outcomes of interest in part A include cough symptom score, time to cough relief and TCM curative effect. Cough relief is defined as both daytime cough symptom score and nighttime cough symptom score ≤ 1 or reduce by one score, for 48 h. TCM curative effect is calculated as percentage of cumulative TCM symptom score reduction (PSSR) estimated between baseline and post-intervention, which is categorized into four grades, complete recovery (PSSR ≥ 95 %), excellent effect (95 % > PSSR ≥ 70 %), modest effect (70 % > PSSR ≥ 30 %) and no effect (PSSR < 30 %). The TCM symptom score system used in the protocol follows the Guidelines for Clinical Research of New Chinese Medicine [26], in which primary and secondary symptoms are given graded scores. The TCM symptom score and TCM signs system is provided in Fig. 2. TCM signs will also be assessed, but not scored. PSSR is calculated according to the following formula:

$$ \mathrm{PSSR}=\left(\frac{symptom\kern0.5em  score\kern0.5em  before\kern0.5em  treatment- symptom\kern0.5em  score\kern0.5em  after\kern0.5em  treatment}{symptom\kern0.5em  score\kern0.5em  before\kern0.5em  treatment}\right)\times 100\% $$

#### Part B

##### Primary outcome

The primary outcome of part B is cough symptom score, which will be assessed across baseline, 6 ± 1 days into the intervention, post-intervention (11 ± 1 days) and 2 days post-intervention if necessary. Simplified cough symptom score (SCSS) recommended by Chinese Medical Association [30] is developed based on the Asthma and Cough Symptom Score Estimation Method [31]. It consists of daytime score and nighttime score, ranging from 0 to 3, respectively. The cough symptom score scale is provided in Table 7. SCSS, which is usually used to determine the frequency and diurnal variation of cough, and also to reflect the quality of life, has been verified as reproducible and well-responsive to treatments [32]. Daytime is arbitrarily defined as 8 a.m. to 8 p.m. and nighttime as 8 p.m. to 8 a.m.

**Table 7:** Cough symptom score scale

Score	Daytime cough symptom score	Nighttime cough symptom score
0	no cough during the day	no cough during the night
1	occasional cough for short periods	cough for short periods before sleep or occasional cough during the night
2	frequent coughing, which did slightly interfere with usual daytime activities	frequent coughing, which did slightly interfere with sleep
3	distressing coughs, which did seriously interfere with usual daytime activities	distressing coughs preventing any sleep

##### Secondary outcomes

Secondary outcomes of part B include visual analogue scale (VAS) [33], cough recovery rate, time to cough resolution, time to cough relief, TCM curative effect and cough-specific quality of life questionnaire (CQLQ) score (Chinese version). The reliability, validity and responsiveness of the Chinese version of CQLQ had been approved [21]. The production and development of this version were conducted followed the international standard guideline [34] through the comprehensive processes of translation, back-translation, committee review and pre-testing technique use. Professor Irwin, the author of the original CQLQ, granted the permission to produce the Chinese version [21].

### Safety outcomes

Safety outcomes in this trial include blood routine test, urine routine test, stool routine test, electrocardiogram, liver function test (ALT, AST, STB, ALP, and γ-GT) and renal function test (Bun and Cr). These biological parameters will be monitored both before and after trial intervention. Urine pregnant test (UPT) is supposed to be done before enrolling a female patient of child-bearing age.

### Data management

Completed CRFs will be reviewed by both chief investigators and a qualified research monitor delegated by Beijing Qihuang Medicine Clinical Research Center. CRFs will be delivered for data management. All study data will be entered in computerized database using EpiData 3.1 software by two data mangers independently, and results will be cross checked for accuracy and consistency. Data entered into the database can be accessible only through authorized identification code and password. Moreover, missing data or false data, if there is any, will be detected by designed application program and recorded by data managers, who are qualified to recheck the original CRFs to ensure the correctness, completeness, and consistency of entered data. The database would be locked after data input. Original CRFs will be kept on file in a secure manner at individual participating sites by chief investigators.

### Statistical analysis

Beijing Qihuang Medicine Clinical Research Center is in charge of data statistical analysis. The statistical analysis will be masked. A group of professional statisticians who are independent of all the other process of the study would perform the statistical analysis. Full Analysis Set (FAS) would be applied for all participants who have been randomly allocated and have taken QFGKG or placebo. Non-completers who never use any test drugs will be excluded. Missing values of primary variations will be imputed using the last observation carried forward (LOCF) approach according to intention-to-treat (ITT) principle. Cases in Per Protocol Set (PPS) are those who thoroughly adhere to the protocol without absence of baseline characteristics. Analysis of primary outcome and curative effect will be carried out using FAS approach and PPS approach. Safety Analysis Set (SS) includes all randomized patients who have accomplished at least one study visit. Participating centers are required to sum up participants number in each set and list participants who are removed from PPS. The Chi-square test or Fisher’s exact test will be performed for the categorical variables, and the Student’s t-test will be used for continuous normally distributed variables. For data that is not normally distributed, intra-group or inter-group differences before and after treatment will be analyzed by Wilcoxon rank sum test. The proportion of patients with adverse events in two groups will be compared using the chi-square test or Fisher’s exact test. The statistical significance level is set at *P* < 0.05 and relative risk (RR) with corresponding 95 % confidence interval (CI) to compare dichotomous variables will be calculated. Table 8 shows the method of analysis for specific outcomes.

**Table 8:** Outcomes and methods of analysis

Outcome/variable	Hypothesis	Measures	Methods of analysis
Baseline balance test		Quantitative outcomes (age, disease duration, body temperature, heart/respiratory rate, blood pressure)	T-test/Wilcoxon rank sum test
		Qualitative outcomes (gender, marriage, race, previous treatment)	Chi-squared test/Fisher exact test/rank-sum test
Adherence at post-intervention		Percent and cases of adherence in previous 10/14 days < 80 %, > 120 % and 80 %-120 %	Chi-squared test/Fisher exact test
Concomitant treatments		Percent and cases of concomitant treatments	Chi-squared test/Fisher exact test
Intervention duration		Formula: the last day using test drugs-the first day using test drugs + 1	T-test/Wilcoxon rank sum test
Primary			
Time to cough resolution	improvement occurred		Kaplan-Meier method/Log-rank test
Cough symptom score	improvement occurred		T-test/Wilcoxon rank sum test
Secondary			
VAS score	improvement occurred		T-test/Wilcoxon rank sum test
TCM symptom score	improvement occurred		T-test/Wilcoxon rank sum test
TCM curative effect	improvement occurred	Percent and cases of four grades: complete recovery, excellent effect, modest effect and no effect	Cochran-Mantel-Haenszel Chi-squared test
CQLQ score	improvement occurred	Questionnaire	T-test/Wilcoxon rank sum test
Cough disappearance rate	improvement occurred		CMH Chi-squared test
Time to cough relief	improvement occurred		Kaplan-Meier method/Log-rank test

### Ethics

This trial is conducted in accordance with the ethical and scientific principles that originate from the Declaration of Helsinki and GCP [35] and local regulations (http://www.sda.gov.cn/ypzcgl/ml.htm). The trial has been authorized by CFDA (Clinical Trial Approval No. 2010 L00279). The current protocol and written informed consent have been approved by the Ethics Committee of Clinical Trials and Biomedicine of West China Hospital of Sichuan University. Until the required quota of participants is reached, all potential patients can join in this trial through two ways, advertisement and review of health records by respiratory physicians. The recruitment advertisement has also been approved by the ethics committee. Participants are promised to be paid 200 RMB as financial incentive for enrollment when the whole trial is finished, and they will be informed detailed information about this trial regarding objectives, procedure, potential risks and benefits, and their rights and obligations prior to enrollment. An informed consent that is written in easy-to-understand language will be given to participants. Patients’ full understanding of the protocol and contentment to every response from physicians must be ensured. Interventions will not be initiated until the written informed consent is obtained from all participants. Physicians are required to give their contact details to each patient recruited. As regards secrecy and confidentiality, data of every single patient will be recorded anonymously, and any information that is suspected to reveal patients’ identification will be concealed. Patients’ confidentiality will be preserved at all times during the whole trial and their medical records will definitely be properly archived in National Drug Clinical Trial Institution, not disclosed to any third party.

## Discussion

Postinfectious cough, a common but not widely recognized and understood disease, imposes a great burden on both physical and mental health of patients. Exploring an effective treatment for PIC is an urgent necessity. TCM is a structured intervention that has shown some promise in the management of persistent cough. A growing number of clinical trials are studying the effect of TCM on PIC. But unfortunately, existing unconvincing evidence makes it awkward to interpret findings in a consistent manner or confirm a conclusion. Although our previous phase II trial is the first study using TCM to treat PIC, some limitations still exist [20], such as the absence of follow-up. This trial aims to fill this gap by undertaking a well-designed multicentre, placebo-controlled, double-blind randomized trial to evaluate the efficacy and safety of a TCM formula, *Qing-Feng-Gan-Ke-Granules*. Moreover, this trial will be an outstanding supplement to this area by providing tangible benefits for PIC research.

In this trial, we conduct two simultaneous study parts that are mutually independent but not mutually exclusive. We aim to not only propose a new effective remedy for PIC but also practice a novel methodology for clinical trials, that is “one study, one primary outcome”. As we all know, a well designed clinical trial is always expected to answer multiple main clinical questions. Nevertheless, sometimes it is unreasonable to assess multi-primary outcomes in one study. In this trial, we intend to investigate the effectiveness of QFGKG on different clinical outcomes. In this case, it may be more convincing and persuasive to study different groups of patients and conduct different intervention durations to achieve exclusive primary outcome by separating the original trial into study parts. We believe that if considering the results of the two study parts as a whole, it would be helpful not only for figuring out ongoing objectives but also for clarifying further implications. Therefore, we assume that this novel design contributes to maximized efficacy, lower cost, a better risk-benefit ratio of treatment and more convincing results with strong application value and may be recommended for further clinical intervention trials. This is the most all-important distinguishing feature of this trial. Another breakthrough in this protocol is that we highlight the importance of two clinical related outcomes, time to cough resolution and quality of life, which are seldom assessed in previous studies [14].

Several limitations and drawbacks of this study are as follows. Firstly, since we are going to conduct different intervention durations in the two parts, some patients with relatively mild cough would be treated for 14 days while those with relatively severe cough will be treated for 10 days, which is paradoxical and against common clinical practice. Secondly, although a careful knowledge of medical history and physical examination including serologic tests may provide clues to diagnosis, the diagnosis of PIC still largely depends on clinical exclusion [5]. Therefore, the possibility of misdiagnosis cannot be ruled out. Thirdly, an objective cough measurement is not included in this study. Many video and sound recording surveillance have been used in clinic to objectively monitor the cough frequency and severity [36–39]. However some studies reported a poor relationship between cough frequency and cough symptom score [37–39]. Furthermore, although operating a manual recording instrument is easy for patients, it requires a good compliance, otherwise, considerable errors could be induced. In addition, how to effectively and sensitively distinguish and filter the external vocal interference, and also record the weak electromyographic signals of pulmonary muscles are still the technical bottlenecks for the development of 24 h cough monitors. Besides, the expensive cost also limits the use of cough recording surveillance. For the above reasons, objective cough measurements are not included in our study. Fourthly, the lack of data from old people over the age of 65 fails to provide information about the efficacy and safety of QFGKG in this group of patients.

### Trial status

The recruitment process was initiated from May 01, 2013 and until now, a total of 318 participants have been included.

## Abbreviations

ALP: Alkaline Phosphatase
ALT: Alanine aminotransferase
AST: Aspartate aminotransferase
BPT: bronchial provocation test
BUN: Blood urea nitrogen
CMH: Cochran-Mantel-Haenszel
CQLQ: cough-related quality of life questionnaire
Cr: Creatinine
CR: chest radiography
CRF: case report form
ECG: Electrocardiogram
FAS: Full Analysis Set
GCP: good clinical practice
QFGKG: Qing-Feng-Gan-Ke Granules
CFDA: China Food and Drug Administration
SS: Safety Analysis Set
PPS: Per Protocol Set
TCM: traditional Chinese medicine
UPT: urine pregnant test
URTI: upper respiratory tract inflammation
γ-GT: γ-Glutamyl Transpeptidase
